# Dissecting Holistic Metabolic Acclimatization of *Mucor circinelloides* WJ11 Defective in Carotenoid Biosynthesis

**DOI:** 10.3390/biology13040276

**Published:** 2024-04-18

**Authors:** Fanyue Li, Roypim Thananusak, Nachon Raethong, Junhuan Yang, Mingyue Wei, Xingtang Zhao, Kobkul Laoteng, Yuanda Song, Wanwipa Vongsangnak

**Affiliations:** 1 Interdisciplinary Graduate Programs in Bioscience, Faculty of Science, Kasetsart University, Bangkok 10900, Thailand; lifanyue.f@ku.th; 2 Colin Rateledge Center for Microbial Lipids, School of Agricultural Engineering and Food Science, Shandong University of Technology, 266 Xincun West Road, Zibo 255000, China; 3 Omics Center for Agriculture, Bioresources, Food, and Health Kasetsart University (OmiKU), Bangkok 10900, Thailand; roypim.tha@ku.th; 4 Institute of Nutrition, Mahidol University, Nakhon Pathom 73170, Thailand; nachon.rae@mahidol.ac.th; 5 Department of Food Sciences, College of Food Science and Engineering, Lingnan Normal University, Zhanjiang 524048, China; yangjh@lingnan.edu.cn; 6 College of Ecology, Resources and Environment, Dezhou University, Dezhou 253000, China; weimy09@foxmail.com; 7 Shandong Key Laboratory of Biophysics, Institute of Biophysics, Dezhou University, Dezhou 253023, China; longhuxingsheng@163.com; 8 Industrial Bioprocess Technology Research Team, Functional Ingredient and Food Innovation Research Group, National Center for Genetic Engineering and Biotechnology (BIOTEC), National Science and Technology Development Agency (NSTDA), Pathum Thani 12120, Thailand; kobkul@biotec.or.th; 9 Department of Zoology, Faculty of Science, Kasetsart University, Bangkok 10900, Thailand

**Keywords:** carotenoids, microbial lipids, *Mucor circinelloides*, reporter metabolites, transcriptome

## Abstract

**Simple Summary:**

The lipid yield of *Mucor circinelloides* WJ11 has been much studied for industrial production improvement. In this study, the *carRP* gene responsible for the production of carotenoids was knocked out, which resulted in the interruption of the carotenoid production pathway and a simultaneous impact on lipid production. Further, an integrative transcriptome and genome-scale metabolic model-driven analysis was conducted, which provides insights into the coordinated relationship between carotenoid and fatty acid biosynthesis in *M. circinelloides*. The findings can be used to design efficient *M. circinelloides* cell factories.

**Abstract:**

*Mucor circinelloides* WJ11 is a lipid-producing strain with industrial potential. A holistic approach using gene manipulation and bioprocessing development has improved lipid production and the strain’s economic viability. However, the systematic regulation of lipid accumulation and carotenoid biosynthesis in *M. circinelloides* remains unknown. To dissect the metabolic mechanism underlying lipid and carotenoid biosynthesis, transcriptome analysis and reporter metabolites identification were implemented between the wild-type (WJ11) and *ΔcarRP* WJ11 strains of *M. circinelloides*. As a result, transcriptome analysis revealed 10,287 expressed genes, with 657 differentially expressed genes (DEGs) primarily involved in amino acid, carbohydrate, and energy metabolism. Integration with a genome-scale metabolic model (GSMM) identified reporter metabolites in the *ΔcarRP* WJ11 strain, highlighting metabolic pathways crucial for amino acid, energy, and nitrogen metabolism. Notably, the downregulation of genes associated with carotenoid biosynthesis and acetyl-CoA generation suggests a coordinated relationship between the carotenoid and fatty acid biosynthesis pathways. Despite disruptions in the carotenoid pathway, lipid production remains stagnant due to reduced acetyl-CoA availability, emphasizing the intricate metabolic interplay. These findings provide insights into the coordinated relationship between carotenoid and fatty acid biosynthesis in *M. circinelloides* that are valuable in applied research to design optimized strains for producing desired bioproducts through emerging technology.

## 1. Introduction

Oleaginous filamentous fungi can accumulate oil to more than 20% of their biomass [1,2,3,4,5]. *Mucor circinelloides* is one of the oleaginous species and has been considered a model organism for studying microbial lipid and carotenoid production due to its metabolic capability to produce high levels of polyunsaturated fatty acids (PUFAs), particularly γ-linolenic acid (GLA) and β-carotene, with nutritional benefits [6,7,8]. Among six different genera of oleaginous fungi, including *Thamnidium*, *Cunninghamella*, *Rhizopus*, *Mucor*, *Mortierella*, and *Gongronella*, *M. circinelloides* was found to be the most efficient in producing GLA, with 22.3 mg GLA/g dry cell weight (DCW) [9]. Thus, it has been commercially used to produce oil rich in GLA [1,10,11]. As a source of β-carotene, *M. circinelloides* also showed to be an excellent filamentous fungi for accumulating up to 698.4 ± 3.68 μg/g of β-carotene [8]. The synthesis of different natural commercially important PUFAs and carotenoids with a broad range of substrate utilization by *M. circinelloides* [12] has led to the consideration of this organism rather than oleaginous yeast, e.g., *Rhodotorula* spp. [13] and *Yarrowia lipolytica* [14], as a potent source having industrial interest.

Recently, the genome sequence of *M. circinelloides* WJ11 has been published, revealing the key genes related to the lipid-accumulating process [15]. By comparing the WJ11 genome to the low-lipid-producing strain CBS277.49, unique genes involved in carbohydrate and lipid metabolism were identified, postulating that the WJ11 strain might provide additional NADPH for lipid accumulation [15,16]. Proteomic studies showed that the expression of glutamine synthetase involved in ammonia assimilation was upregulated when nitrogen was depleted, while the expression of proteins involved in amino acid biosynthesis was downregulated. At the same time, the expression of proteins involved in the tricarboxylic acid (TCA) cycle was downregulated, indicating that cells promoted the biosynthesis of fatty acids by coordinating central carbon metabolism when nitrogen was restricted [17].

As earlier described, *M. circinelloides* has also been reported to produce a high amount of β-carotene under light exposure in contrast to the culture grown under dark conditions [18]. Research exploring the enhanced production of both lipids and carotenoids in *M. circinelloides* has been conducted [8]. It has been stated that *M. circinelloides* also contains a minor amount of zeaxanthin [19], in contrast to the oleaginous yeast *Rhodotorula glutinis*, which only produces carotenoids [20]. Under nitrogen-exhausted and excess glucose conditions, there was evidence that the acetyl-CoA precursor was shared for the lipogenesis and carotenogenesis pathways in *M. circinelloides*, which seems to be a reverse relationship [21,22]. Studies have reported that under nitrogen starvation, oleic acid mainly accumulates in triacylglycerol, and the accumulation of astaxanthin monoester shows a linear relationship [23]. It seems that the synthesis of fatty acids is related to the accumulation of carotenoids [24]. Presumably, lipid biosynthesis and accumulation might be enhanced by eliminating carotenogenesis. A single gene (*carRP*) encoding for a protein with bifunctional enzyme activity, i.e., lycopene cyclase and phytoene synthase, responsible for carotenogenesis was identified in *M. circinelloides* [25] and has been postulated to be a target gene for enhancing lipid accumulation by the gene deletion approach [26]. However, systematic regulation of lipid accumulation and carotenoid biosynthesis in *M. circinelloides* should be addressed to offer a precise manipulation strategy. Therefore, this study aimed to investigate the global metabolic changes of *M. circinelloides* WJ11 defective in carotenoid biosynthesis (*ΔcarRP* WJ11 strain) in the active growth phase. A comparative transcriptome analysis was implemented between the wild-type (WJ11) and *ΔcarRP* WJ11 strains of *M. circinelloides* under such conditions. A genome-scale metabolic model (GSMM)-driven analysis was also performed with the integration of differentially expressed gene (DEG) data to identify the key metabolites associated with the lipid biosynthetic pathway [16,27]. We also postulate the vital metabolic changes in other pathways of the *ΔcarRP* WJ11 strain to maintain cell homeostasis through the cooperation of the transcriptional controls. This study provides a perspective for designing efficient cell factories of *M. circinelloides*, which is valuable knowledge in industrial biotechnology.

## 2. Materials and Methods

### 2.1. Fungal Strains and Cultivations

*M. circinelloides* strain WJ11 (wild-type) [6] was used as a reference in this study. *M. circinelloides* WJ11 defective in the *carRP* gene (*ΔcarRP* WJ11) was employed for investigating lipid and carotenoid biosynthesis, with reference to the Mc2075 strain with knock out of the *carRP* gene performed through homologous recombination by using the designed pMAT2075 plasmid [28].

For fungal cultivation, seed preparation of each strain was performed by growing the cells in K&R medium, one liter of which consisted of 30 g of glucose, 1.5 g of MgSO_4_·7H_2_O, 3.3 g ammonium tartrate, 7.0 g of KH_2_PO_4_, 2.0 g of Na_2_HPO_4_, 1.5 g of yeast extract, 0.1 g of CaCl_2_·2H_2_O, 8 mg of FeCl_3_·6H_2_O, 1 mg of ZnSO_4_·7H_2_O, 0.1 mg of CuSO_4_·5H_2_O, 0.1 mg of Co(NO_3_)_2_·6H_2_O, and 0.1 mg of MnSO_4_·5H_2_O [29], under shaking at 150 rpm and 28 ± 1 °C for 24 h. The seed culture was then inoculated into a 2 L fermenter (BioFlo/CelliGen115; New Brunswick Scientific, NJ, USA) with a 1.5 L working volume of the medium broth containing 80 g/L glucose and 2 g/L ammonium tartrate. The cultivation conditions were constantly controlled, including culture temperature of 28 ± 1 °C, pH of 6.0, stirring speed of 700 rpm, and airflow rate of 1 vvm. The culture samples were collected for biomass and metabolite determination at different time points. All experiments were performed in three biological replicates.

### 2.2. Biomass and Metabolite Determination

The harvested mycelial samples were dried by using a freeze dryer at −30 °C for 2 days. Dried samples were then weighted and subjected to calculate biomass concentration represented as dry cell weight (DCW). Total lipids of dried mycelia were extracted by using a chloroform/methanol (2:1, *v*/*v*) solution [30] and then methylated with 4 mol/L methanolic HCl at 60 °C for 3 h. The fatty acid methyl esters were then extracted with n-hexane and analyzed by a gas chromatography flame ionization detector (GC-FID) equipped with a 30 m × 0.32 mm DB-WAXETR column (0.25 μm in film thickness) [6]. The GC analysis was performed at 120 °C for 3 min, ramped to 200 °C at 5 °C per min, ramped to 220 °C at 4 °C per min, and held for 2 min. Pentadecanoic acid (15:0) was used as an internal standard for calculating the concentration of individual fatty acids by using their chromatographic areas.

For the carotenoid analysis, a 50 mg dried mycelial sample was extracted with 800 μL of acetone and then mixed using a vortex. This extraction step was then repeated until the mycelial pellet was colorless. Extracts were partitioned with an equal volume of 10% diethyl ether in petroleum ether. Acetone and petroleum ether were removed by distilled water and nitrogen gas, respectively. The pigment extract was resuspended in 700 μL of tetrahydrofuran supplemented with 250 ppm of butylated hydroxytoluene and subjected to high-performance liquid chromatography (HPLC) as described in [8].

### 2.3. RNA Extraction, Transcriptome Sequencing, and Quality Analysis

**Figure 1:**
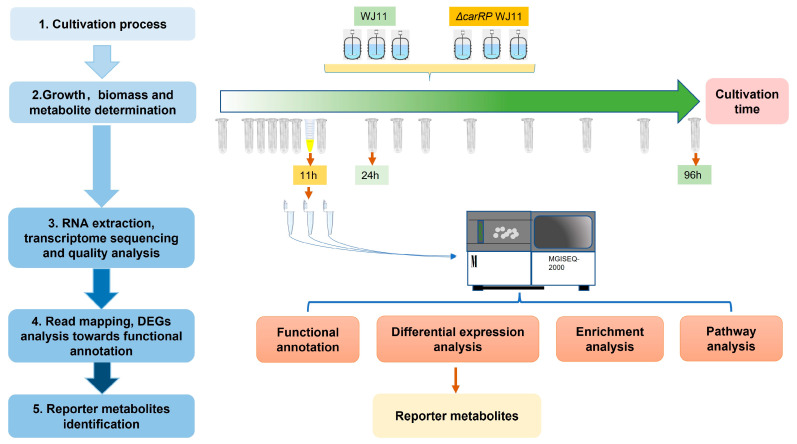
Systematic workflow of this study by integrative analysis of transcriptome data of *M. circinelloides* strains WJ11 and *ΔcarRP* WJ11.

To perform transcriptome sequencing (Figure 1), the mycelial cells of *M. circinelloides* WJ11 and *ΔcarRP* WJ11 strains grown in a fermenter for 11 h of cultivation time under dark conditions were harvested, immediately frozen in liquid nitrogen, and then stored at −80 °C before RNA extraction. Total RNA was extracted by using RNeasy Plant Mini Kit (Qiagen), and the quality and concentration of total RNA were determined by using an Agilent 2100 bioanalyzer. Further, the cDNA library construction and transcriptome sequencing of three biological replicates of each strain were performed by using the MGISEQ-2000RS platform.

To examine raw RNA-Seq data, they were processed by using FastQC [31]. To gain clean reads with high-quality data, joint contamination, unknown bases (N > 5%), and low-quality reads were filtered, and adapters sequences were trimmed by using the SOAPnuke program [32]. The processed RNA-Seq data were kept in FASTQ format and were deposited in the NCBI Sequence Read Archive (SRA) under BioProject accession number PRJNA1013727 (BioSamples SAMN37309028, SAMN37309029, SAMN37309030, SAMN37309031, SAMN37309032, and SAMN37309033).

### 2.4. Read Mapping and DEG Analysis for Functional Annotation

For read mapping, the clean reads of RNA-Seq data were mapped to the *M. circinelloides* 1006PhL genome [33] by using HISAT (Hierarchical Indexing for Spliced Alignment of Transcripts) [34] and the Bowtie2 program [35,36]. It is noted that *M. circinelloides* 1006PhL was selected in this study because it has a high-coverage genome according to NCBI and has thus been widely used as the reference genome for several comparative genomics and genetic studies [37,38]. At first, clean reads were efficiently calibrated by HISAT. After that, we used HISAT to align the clean reads to the reference genome sequence. Bowtie2 was further used to align clean reads to reference gene sequences. The genes with mapped reads were subjected to calculation of FPKM (fragment per kilobase of transcript per million mapped reads), a normalized gene expression value based on RNA-Seq data. The expressed genes were indicated by an FPKM value ≥ 1. The differential expression analysis across the two strains was performed by using DESeq2 in the R package [39,40]. The list of significant genes under |log2 (fold change)| ≥ 1 and Q-value < 0.05 were then identified. The annotated protein functions of the significant genes were retrieved from the Kyoto Encyclopedia of Genes and Genomes (KEGG), Gene Ontology (GO), and the Non-Redundant Protein Sequence Database (NR). KEGG and GO pathway enrichment analyses were also performed by using the hypergeometric test [41,42,43,44].

### 2.5. Reporter Metabolites Identification Based on the Genome-Scale Metabolic Model of M. circinelloides WJ11 (iNI1159)

An earlier GSMM of *M. circinelloides* (*i*NI1159) [13] was employed to identify the reporter metabolites. The expressed genes were matched with the list of gene–metabolite sets by using the EC number ortholog parsing method. Next, the matched gene–metabolite sets as a scaffold were integrated with the list of DEGs obtained from the comparative transcriptome result of the two strains (WJ11 and *ΔcarRP* WJ11) for identifying the key metabolites and related metabolic pathways by using the consensus gene set enrichment analysis for reporter metabolites identification with the piano R package [45,46]. The metabolite with a distinct up-directional *p*-value < 0.05 was identified as a reporter metabolite.

## 3. Results and Discussion

### 3.1. Comparative Growth Profiles and Targeted Metabolite Traits of WJ11 and ΔcarRP WJ11 Strains

The comparative growth characteristics and the production of lipids and carotenoids of the wild-type (WJ11) and the gene-knock-out strain (*ΔcarRP* WJ11) are shown in Table 1 and Supplementary Table S1. The WJ11 culture had a biomass titer higher than that of the *ΔcarRP* WJ11 culture (Table 1), similarly to previous studies [6]. The maximum specific growth rate of the WJ11 culture (μ_max_ of 0.765 ± 0.5 h^−1^) was higher than that of *ΔcarRP* WJ11 (μ_max_ of 0.665 ± 0.17 h^−1^) (Table 1), and the biomass titer of the gene-knock-out strain was lower than that of the wild-type. Fatty acids in DCW (g/g, %) were not significantly different between these two strains. The carotenoid content in WJ11 was higher than that in *ΔcarRP* WJ11 (Table 1). The biomass titer (DCW) of the wild-type culture was higher than that of the gene-knock-out strain, which seems to be indicative of diauxic growth. Diauxic growth has been reported in the oleaginous fungus *Mucor rouxii* [47], with evidence indicating that fungal cells might utilize the secreted ethanol as a secondary carbon source for secondary growth. There was an increase in biomass rich in β-carotene for the WJ11 culture during the secondary growth stage, which was the lipid-accumulating phase [48,49]. In contrast, the biomass productivity was lower in the gene-knock-out strain, even though it could accumulate fatty acids in the cells at a similar level to the WJ11 culture (Table 1). The carotenoid content within the cells of the knocked-out *carRP* gene strain exhibited a significant decrease, as expected due to the elimination of one of the structural genes of the carotenogenesis pathway.

### 3.2. Transcriptome Data and DEGs across Pairwise Comparisons of WJ11 and ΔcarRP WJ11 Cultures

Due to the discrimination in phenotypic characteristics between the wild-type and disruptant strains in the active growth phase, which is the stage when metabolism is active [50], we collected the cells after 11 h of cultivation time under dark conditions, which had the maximum specific growth rates (Table 1), to investigate their global transcriptional responses. The transcriptome analysis of WJ11 and *ΔcarRP* WJ11 strains showed that raw reads were obtained at an average sequencing depth of 43.82 million reads. After removing the adaptor, low-quality sequences, and read pollution, clean reads were retrieved with average sequencing depth values of 43.18 and 43.12 million reads for the WJ11 and *ΔcarRP* WJ11 strains, respectively. The sequencing quality rates of WJ11 and *ΔcarRP* WJ11 strains were 96.78% and 96.93%, respectively (Table 2). Consequently, they were mapped through the *M. circinelloides* 1006PhL genome, resulting in the average total mapped reads of 93.49% and 93.86% for the WJ11 and *ΔcarRP* WJ11 strains, respectively. The expressed genes for the WJ11 and *ΔcarRP* WJ11 cultures were 10,063 and 10,162, respectively (Supplementary Table S2).

**Figure 2:**
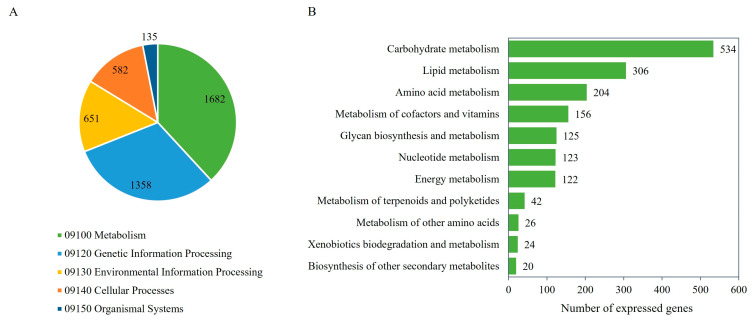
The number of the expressed genes of the *M. circinelloides* WJ11 and *ΔcarRP* WJ11 cultures and their KEGG functional classification. (**A**) The functional annotation of the expressed genes. (**B**) The metabolic functional categories of the expressed genes.

As listed in Table 2, 10,287 expressed genes as protein-encoding genes were identified, of which 10,283 (99.96%) were annotated with protein functions according to the NR, GO, or KEGG database [41,51,52,53]. Of the 10,283 genes, the putative functions could be predicted based on NR (10,280 genes), GO (7813 genes), and KEGG Orthology (KO) (7273 genes). A list of annotated genes and putative functions according to the different protein databases used in this study is listed in Supplementary Table S3. Figure 2A shows five major functional categories of the expressed genes based on the KEGG database, which included metabolism (1682 genes), genetic information processing (1358 genes), environmental information processing (651 genes), cellular processes (582 genes), and organismal systems (135 genes). For the metabolic category, the expressed genes related to carbohydrate metabolism (534 genes), lipid metabolism (306 genes), amino acid metabolism (204 genes), metabolism of cofactors and vitamins (156 genes), glycan biosynthesis and metabolism (125 genes), nucleotide metabolism (123 genes), energy metabolism (122 genes), metabolism of terpenoids and polyketides (42 genes), metabolism of other amino acids (26 genes), xenobiotic biodegradation and metabolism (24 genes), and biosynthesis of other secondary metabolites (20 genes) were identified (Figure 2B and Supplementary Table S4).

**Figure 3:**
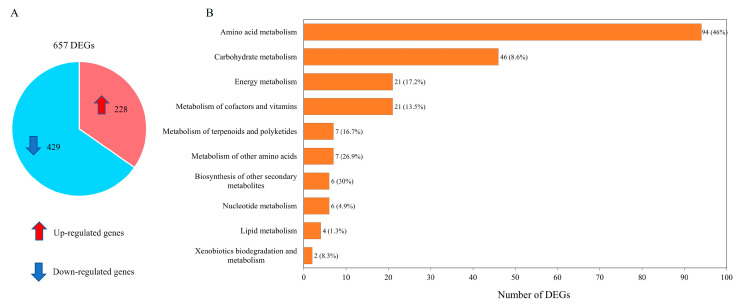
Differentially expressed genes (DEGs) between *M. circinelloides* WJ11 and *ΔcarRP* WJ11 strains. (**A**) A pie chart shows the number of DEGs. (**B**) The enrichment analysis of DEGs based on KEGG annotation. Note in Figure 3B: The front number represents the identified number of DEGs, and the percentage in parenthesis represents the proportion of DEGs identified from all the expressed genes existing in each metabolic pathway.

Transcriptome data were organized into pairwise comparisons between WJ11 and *ΔcarRP* WJ11 strains. By using the thresholds of |log2 (fold change)|  ≥  1 with Q-value ≤  0.05 in pairwise comparisons, a total number of 657 differentially expressed genes (DEGs) was identified, as illustrated in Figure 3A and Supplementary Table S5. It was shown that 228 upregulated and 429 downregulated DEGs were obtained when comparing the *ΔcarRP* WJ11 and WJ11 strains (Figure 3A). To explore the critical attributes in the transcriptional regulation of WJ11 and *ΔcarRP* WJ11 cultures, a functional enrichment analysis was performed by using the DEG metabolic pathways according to a *p*-value < 0.09 (Figure 3B and Supplementary Table S6). The highest number of DEGs (94 genes) was found in amino acid metabolism, whereas the lowest number of DEGs (2 genes) was detected in xenobiotic biodegradation and metabolism.

The majority of DEGs enriched in amino acid metabolism were found in alanine, aspartate, and glutamate metabolism (14 genes), followed by phenylalanine, tyrosine, and tryptophan biosynthesis (12 genes); arginine biosynthesis (12 genes); and arginine and proline metabolism (11 genes). Other pathways with a high number of DEGs included lysine degradation; glycine, serine, and threonine metabolism; cysteine and methionine metabolism; and valine, leucine, and isoleucine degradation (listed in Supplementary Table S6). Table 3 shows list of DEGs that encoded enzymes involved in various metabolic reactions related to amino acid metabolism, nitrogen metabolism, central carbon metabolism, and fatty acid and carotenoid biosynthesis. In the *ΔcarRP* WJ11 strain, for instance, the significant gene encoding for ornithine carbamoyltransferase was upregulated by 5.59 times compared with the WJ11 strain. The enzymes carbamoyl-phosphate synthase (EC:6.3.5.5), argininosuccinate lyase (EC: 4.3.2.1), and argininosuccinate synthase (EC: 6.3.4.5), responsible for producing carbamoyl phosphate, fumarate and arginine, and succinate, in the *ΔcarRP* WJ11 strain were also transcriptionally upregulated by3.14, 3.29, and 3.28 times, respectively. Genes involved in chorismate production, such as phosphoheptulonate synthase (EC: 2.5.1.54), were also significantly upregulated in the *ΔcarRP* WJ11 strain. The transcriptional upregulation of the enzyme-encoding genes responsible for NADP generation, such as nitrite reductase (EC: 1.7.1.4) and pyrroline-5-carboxylate reductase (EC: 1.5.1.2), was additionally found in the *ΔcarRP* WJ11 strain.

In carbohydrate metabolism, the 46 DEGs were mainly involved in glycolysis and gluconeogenesis, pyruvate metabolism, propanoate metabolism, glyoxylate and dicarboxylate metabolism, ascorbate and aldarate metabolism, and fructose and mannose metabolism. Glycolysis involves the conversion of fructose bisphosphate (FBP) into glyceraldehyde 3-phosphate (G3P) by using the enzyme fructose bisphosphate aldolase (EC: 4.1.2.13). However, in the *ΔcarRP* WJ11 strain, this enzyme was found to be downregulated, leading to a decrease in G3P production. Moreover, pyruvate kinase (EC: 2.7.1.40), which is responsible for the production of pyruvate, was also slightly downregulated in the *ΔcarRP* WJ11 strain. This resulted in a reduction in pyruvate production, which could have implications for the overall efficiency of glycolysis in the knock-out strain compared with WJ11 [6]. Remarkably, the isocitrate dehydrogenase (EC: 1.1.1.42) of the *ΔcarRP* WJ11 strain was upregulated by 1.94-fold, thus allocating more energy supply and carbon flux to amino acid biosynthesis.

Twenty-one DEGs involved in energy metabolism, including nitrogen metabolism, methane metabolism, and oxidative phosphorylation, were identified. In the *ΔcarRP* WJ11 strain, four genes encoding nitrate reductase, nitrite reductase, glutamate dehydrogenase, and glutamine synthetase were upregulated. These enzymes can reduce nitric acid to nitrous acid, reducing the production of fermenting acid and thereby maintaining cell viability [54]. These enzymes are key enzymes involved in nitrogen assimilation and are important for maintaining intracellular nitrogen balance [55].

In addition, several enzymes were found to be upregulated in the *ΔcarRP* WJ11 strain, such as acetylglutamate kinase (EC: 2.7.2.8) and carbamoyl-phosphate synthase (EC: 6.3.5.5), which are two enzymes that use ATP for catalysis. Glutaryl-CoA dehydrogenase (EC: 1.3.8.6) was also significantly upregulated, which could potentially provide more acetyl-CoA for the TCA cycle. Upregulation of hydroxyphenylpyruvate dioxygenase (EC: 1.13.11.27) in the *ΔcarRP* WJ11 strain was also observed, suggesting that the fungal cell might generate other precursors for the citrate cycle. This enzyme catalyzes the catabolism of tyrosine, producing fumaric acid, which can enter the TCA cycle [56]. Acetolactate synthase (EC: 2.2.1.6) and branched-chain amino acid aminotransferase (EC: 2.6.1.42) were also upregulated, indicating that the *ΔcarRP* WJ11 strain might use more pyruvate outside the mitochondria to synthesize valine.

However, the downregulation of acetyl-CoA synthetase (EC: 6.2.1.1) and ATP citrate lyase (EC: 2.3.3.8) might lead to a reduction in the acetyl-CoA pool for fatty acid synthesis [28]. Downregulation of fatty acid synthase (FAS1) (EC: 2.3.1.86), involved in long-chain fatty acid synthesis, was also found in *ΔcarRP* WJ11. Two other DEGs that encode for acetyl-CoA carboxylase (EC: 6.4.1.2), a crucial precursor substance for fatty acid synthesis, were also downregulated in *ΔcarRP* WJ11. Acetyl-CoA carboxylase is a key enzymatic reaction in the first step of fatty acid synthesis [57]. The overexpression of acetyl-CoA carboxylase can increase the total fatty acid yield of non-oleaginous yeast, e.g., *Hansenula polymorpha* and *Mucor* spp. [58,59]. This indicates that *carRP* gene deletion impacts fatty acid synthesis through the downregulation of acetyl-CoA carboxylase.

There were seven DEGs related to the metabolism of terpenoids and polyketides, dominated by carotenoid biosynthesis. In the *ΔcarRP* WJ11 strain, for example, phytoene synthase/lycopene beta-cyclase (EC: 2.5.1.32), responsible for generating phytoene, was significantly downregulated by 7.81 times, which is a result of *carRP* gene deletion, leading to defects in phytoene and β-carotene production.

### 3.3. Metabolic Responses of Lipid and β-Carotene Biosynthetic Pathways in WJ11 and ΔcarRP WJ11 Strains

**Figure 4:**
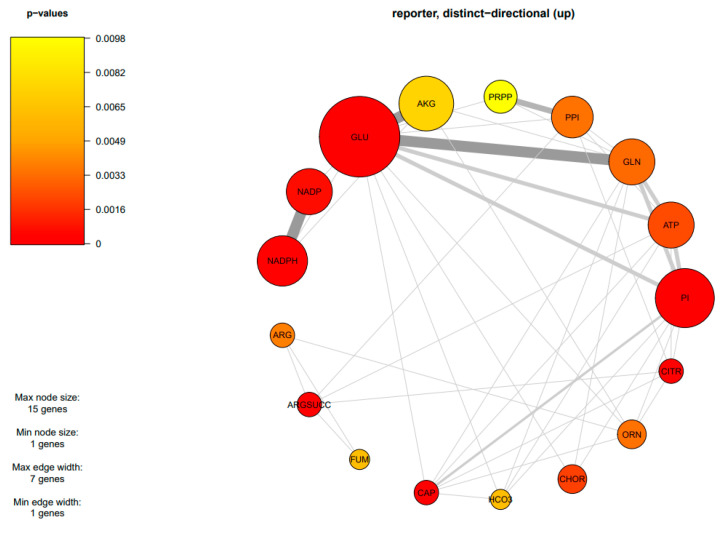
Subnetwork of reporter metabolites of *M. circinelloides* when comparing *ΔcarRP* WJ11 and WJ11 strains. The abbreviations of all metabolite names are in Table 4.

**Figure 5:**
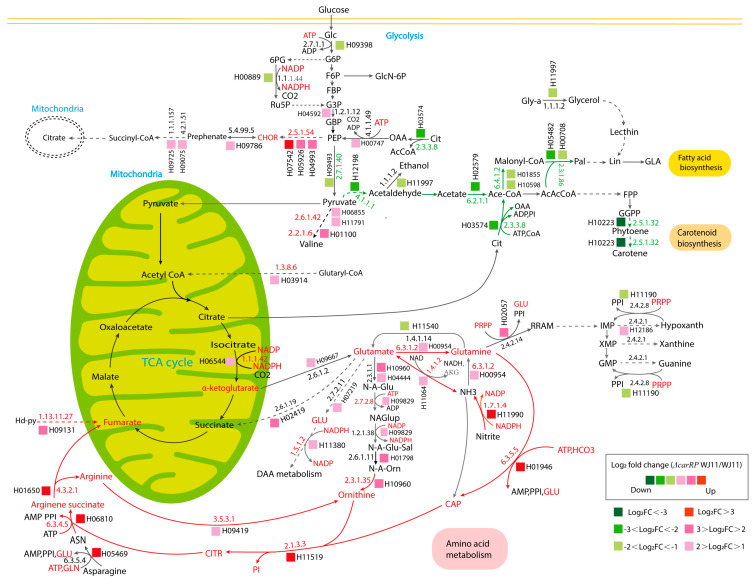
The proposed metabolic routes associated with lipid and β-carotene biosynthetic pathways in *ΔcarRP* WJ11 in comparison with the WJ11 strain. The abbreviations of all metabolite names are in Supplementary Table S8. The abbreviations of all gene IDs are in Supplementary Table S3. A red metabolite name represents a reporter metabolite. Red EC numbers are associated with upregulated genes. The red arrows indicate upregulation routes associated with amino acid metabolism. The green arrows indicate downregulation routes associated with carotenoid and fatty acid biosynthesis. Green EC numbers are associated with downregulated genes.

Due to the finding of DEGs being mostly enriched in amino acid and carbohydrate metabolism, an integrative analysis between transcriptome data and the GSMM of *M. circinelloides* WJ11 (*i*NI1159) was performed to explore the metabolic responses of *ΔcarRP* WJ11 through the transcriptional regulation of significant metabolic genes. As a result, 18 reporter metabolites were identified (Figure 4, Table 4, and Supplementary Table S7). These reporter metabolites are mainly involved in pyrimidine, amino acids, energy, and nitrogen metabolism and some examples are 5-phospho-alpha-D-ribose 1-diphosphate, glutamate, glutamine, arginine, arginine succinate, NADPH, ATP, citrulline, and ornithine. These reporter metabolites were found to be enriched by several upregulated genes associated with the metabolism of arginine and glutamate, and the urea cycle, such as argininosuccinate synthase (HMPREF1544_06810), argininosuccinate lyase (HMPREF1544_01650), arginase (HMPREF1544_09419), glutamate N-acetyltransferase (HMPREF1544_10960), and ornithine carbamoyltransferase (HMPREF1544_11519), as shown in Figure 5 and Table 3. The urea cycle serves a vital role in eliminating excess nitrogen through the production of urea [60,61]. Ornithine carbamoyltransferase is a key player in this process, as it facilitates the conversion of ornithine to citrulline [62]. Additionally, argininosuccinate synthase aids in creating argininosuccinate, which is a building block for arginine [63]. Argininosuccinate lyase then breaks down argininosuccinate into arginine and fumarate. Finally, arginase (EC: 3.5.3.1) hydrolyzes arginine into urea and ornithine, while fumarate enters the TCA cycle [61,64,65]. Consistently, genes responsible for the TCA cycle were upregulated, including the gene for isocitrate dehydrogenase. The upregulation of the isocitrate dehydrogenase gene can also increase the accumulation of α-ketoglutarate, leading to an increase in the amount of carbon entering the TCA cycle (Figure 5). In addition, isocitrate dehydrogenase plays a key role in NADPH formation, which is essential to maintaining the balance of reactive oxygen species (ROS) in the mitochondria. However, excessive accumulation of NADPH can trigger apoptosis caused by an increase in ROS and redox stress. Considering that redox-associated NADPH is a substrate of fatty acid synthase (FAS1) (EC: 2.3.1.86), the downregulation of FAS1 and fatty acid biosynthesis in the *ΔcarRP* WJ11 strain could lead to NADPH accumulation and redox stress. This is consistent with the upregulation of genes involved in converting NADPH to NADP in nitrogen metabolism, such as nitrite reductase (EC: 1.7.1.4) and pyrroline-5-carboxylate reductase (EC: 1.5.1.2), as shown in Figure 5. We hypothesized that the upregulation of amino acid metabolism and urea cycle in the *ΔcarRP* WJ11 strain might favor compensating for the metabolic imbalance caused by a decrease in fatty acid biosynthesis and a defect in the biosynthesis of carotenoid, which has a protective effect on oxidative stress by eliminating excess ROS, quenching singlet oxygen, and promoting better cell growth [66]. Therefore, after knocking out the *carRP* gene, the metabolic balance in the cell is disrupted, leading to reduced production of biomass and carotenoids, as well as slightly reduced lipid production (Table 1).

Moreover, the genes that play a crucial role in generating acetyl-CoA, such as ATP citrate lyase (EC: 2.3.3.8) and acetyl-CoA synthetase (EC: 6.2.1.1), were also observed to be significantly downregulated, as shown in Figure 5. In addition, we also speculate that phytoene synthase/lycopene beta-cyclase (EC: 2.5.1.32) and pyruvate decarboxylase (EC: 4.1.1.1) are positively correlated (Table 3). Therefore, the production of ethanol and acetyl-CoA in the cytoplasm might be decreased due to the absence of phytoene synthase/lycopene beta-cyclase expression and the downregulation of pyruvate decarboxylase in the *ΔcarRP* WJ11 strain, thus leading to no improvement in lipid production, despite acetyl-CoA not being used for carotenoid biosynthesis. Overall, the results highlight a coordinated relationship between carotenoid and fatty acid biosynthesis in oleaginous *M. circinelloides*.

## 4. Conclusions

An integrative approach was employed in the study to analyze the metabolic responses of *M. circinelloides* WJ11 defective in carotenoid biosynthesis. The results indicate that knocking out the *carRP* gene in the WJ11 strain led to an increase in amino acid production and a reduction in carbon allocation towards fatty acid biosynthesis. Downregulation of genes encoding precursors for fatty acid biosynthesis was related to defective carotenoid biosynthesis. This highlights the interdependence between carotenoid and fatty acid biosynthesis, which is useful for developing an optimized strain for producing desired bioproducts by using gene-editing technology.

## Figures and Tables

**Table 1:** Growth and production of fatty acids in DCW and carotenoids in *M. circienlloides* WJ11 and *ΔcarRP* WJ11 strains.

Phenotypic Characteristics	*M. circinelloides* WJ11	*M. circinelloides ΔcarRP* WJ11
Maximum specific growth rate, µ_max_ (h^−1^)	0.765 ± 0.5 ^a^	0.665 ± 0.17 ^a^
Biomass productivity (gDCW L^−1^)	12.95 ± 0.6 ^a^	8.83 ± 1.03 ^b^
Fatty acids in DCW (g gDCW^−1^, %)	35.18 ± 2.18 ^a^	31.45 ± 6.36 ^a^
Carotenoid content (μg gDCW^−1^)	208.99 ± 7.24 ^a^	2.47 ± 0.4 ^b^

Note: Values are means ± SDs (*n* = 3). ^a,b^ Different superscript letters in rows indicate statistically significant differences (*p*-value ≤ 0.05, Tukey’s test). The maximum specific growth rates of *M. circinelloides* WJ11 and *ΔcarRP* WJ11 strains were all obtained from cultivation for 11 h. The highest biomass productivity of *M. circinelloides* WJ11 and *ΔcarRP* WJ11 strains were all obtained from cultivation for 84 h. The highest fatty acids/DCW% of *M. circinelloides* WJ11 and *ΔcarRP* WJ11 strains were all obtained from cultivation for 96 h. The highest carotenoid contents of *M. circinelloides* WJ11 and *ΔcarRP* WJ11 were obtained from cultivations for 48 and 36 h, respectively.

**Table 2:** Mapping results of *M. circinelloides* WJ11 and *ΔcarRP* WJ11 transcriptomes.

Features	WJ11	*ΔcarRP* WJ11
Sequencing depth (million reads)	43.82	43.82
Total clean reads (million reads)	43.18	43.12
Sequencing quality (%)	96.78	96.93
Total mapped reads for genome (%)	93.49	93.86
Total mapped reads for gene (%)	68.84	70.04
Number of expressed genes	10,063	10,162
Total number of expressed genes	10,287	

**Table 3:** List of selected DEGs involved in metabolism.

Gene Name	Functions (EC Number)	Metabolic Pathway	Log_2_FC
HMPREF1544_11519	Ornithine carbamoyltransferase (EC: 2.1.3.3)	Arginine and proline metabolism	5.59
HMPREF1544_06810	Argininosuccinate synthase (EC: 6.3.4.5)	Arginine and proline metabolism	3.28
HMPREF1544_01650	Argininosuccinate lyase (EC: 4.3.2.1)	Arginine and proline metabolism	3.29
HMPREF1544_11380	Pyrroline-5-carboxylate reductase (EC: 1.5.1.2)	Arginine and proline metabolism	1.47
HMPREF1544_09829	Acetylglutamate kinase (EC: 2.7.2.8)	Arginine and proline metabolism	1.50
HMPREF1544_09419	Arginase (EC: 3.5.3.1)	Arginine and proline metabolism	1.83
HMPREF1544_10960	glutamate N-acetyltransferase (EC: 2.3.1.35)	Arginine biosynthesis	2.88
HMPREF1544_01946	Carbamoyl-phosphate synthase (EC:6.3.5.5)	Alanine, aspartate, and glutamate metabolism	3.14
HMPREF1544_07542	Phosphoheptulonate synthase (EC:2.5.1.54)	Phenylalanine, tyrosine, and tryptophan biosynthesis	3.26
HMPREF1544_09131	Hydroxyphenylpyruvate dioxygenase (EC:1.13.11.27)	Phenylalanine, tyrosine, and tryptophan biosynthesis	2.11
HMPREF1544_03914	Glutaryl-CoA dehydrogenase (EC: 1.3.8.6)	Lysine degradation	1.40
HMPREF1544_01100	Acetolactate synthase (EC:2.2.1.6)	Valine, leucine, and isoleucine metabolism	2.09
HMPREF1544_06855	Branched-chain amino acid aminotransferase (EC:2.6.1.42)	Valine, leucine, and isoleucine metabolism	1.27
HMPREF1544_11990	Nitrite reductase (NAD(P)H) (EC: 1.7.1.4)	Nitrogen metabolism	4.35
HMPREF1544_11989	Nitrate reductase (NAD(P)H) (EC:1.7.1.1; 1.7.1.2; 1.7.1.3)	Nitrogen metabolism	4.92
HMPREF1544_11064	Glutamate dehydrogenase (EC:1.4.1.2)	Nitrogen metabolism	1.96
HMPREF1544_00954	Glutamine synthetase (EC:6.3.1.2)	Nitrogen metabolism	1.07
HMPREF1544_09200	Fructose-bisphosphate aldolase (EC:4.1.2.13)	Glycolysis/gluconeogenesis	−1.33
HMPREF1544_09493	Pyruvate kinase (EC:2.7.1.40)	Glycolysis/gluconeogenesis	−1.01
HMPREF1544_02579	Acetyl-CoA synthetase (EC:6.2.1.1)	Pyruvate metabolism	−2.94
HMPREF1544_12198	Pyruvate decarboxylase (EC: 4.1.1.1)	Pyruvate metabolism	−2.08
HMPREF1544_06544	Isocitrate dehydrogenase (EC:1.1.1.42)	Tricarboxylic acid cycle	1.94
HMPREF1544_03574	ATP citrate lyase (EC:2.3.3.8)	Tricarboxylic acid cycle	−2.68
HMPREF1544_05482	Fatty acid synthase (EC:2.3.1.86)	Fatty acid biosynthesis	−2.10
HMPREF1544_01855	Acetyl-CoA carboxylase (EC:6.4.1.2)	Fatty acid biosynthesis	−1.92
HMPREF1544_10598	Acetyl-CoA carboxylase (EC:6.4.1.2)	Fatty acid biosynthesis	−1.62
HMPREF1544_10223	Phytoene synthase/lycopene beta-cyclase (EC:2.5.1.32)	Carotenoid biosynthesis	−7.81

**Table 4:** List of reporter metabolites of *M. circinelloides* when comparing *ΔcarRP* WJ11 and WJ11 strains.

Reporter Metabolite	Up-Directional *p*-Value
Glutamate (GLU)	0.00009999 *
Phosphate (PI)	0.00009999 *
Carbamoyl phosphate (CAP)	0.00009999 *
NADPH	0.00029997 *
Arginine succinate (ARGSUCC)	0.00029997 *
Citrulline (CITR)	0.00029997 *
NADP	0.00059994 *
Chorismate (CHOR)	0.0019998 *
ATP	0.0022998 *
Glutamine (GLN)	0.0031997 *
Diphosphate (PPI)	0.0033997 *
Ornithine (ORN)	0.0033997 *
Arginine (ARG)	0.0037996 *
Fumarate (FUM)	0.0062994 *
HCO_3_	0.0062994 *
α-Ketoglutarate (AKG)	0.0074993 *
5-Phospho-alpha-D-ribose 1-diphosphate (PRPP)	0.009799 *
CO_2_	0.012199

Note: * represents the metabolite with a distinct up-directional *p*-value < 0.01 that was identified as a significant reporter metabolite.

## Data Availability

Raw sequencing data are available in the National Center for Biotechnology Information Sequence Read Archive (NCBI-SRA) repository under BioProject accession number PRJNA1013727 (BioSamples SAMN37309028, SAMN37309029, SAMN37309030, SAMN37309031, SAMN37309032, and SAMN37309033).

## Supplementary Materials

The following supporting information can be downloaded at: https://www.mdpi.com/article/10.3390/biology13040276/s1, Table S1: Growth and β-carotene in *M. circinelloides* WJ11 and *ΔcarRP* WJ11, Table S2: List of expressed genes in *M. circinelloides* WJ11 and *ΔcarRP* WJ11, Table S3: Annotation according to KEGG, NR, and GO, Table S4: List of expressed genes with KEGG annotation, Table S5: List of differentially expressed genes (DEGs) between *M. circinelloides* *ΔcarRP* WJ11 and WJ11 (reference) strains, Table S6: The enrichment analysis of DEGs of *M. circinelloides* based on KEGG annotation, Table S7: List of reporter metabolites with up-directional changes in *M. circinelloides* *ΔcarRP* WJ11, Table S8: Abbreviation of metabolites and genes of *M. circinelloides* in Figure 5.
